# Principles and techniques of imaging in identifying the substrate of ventricular arrhythmia

**DOI:** 10.1007/s12350-015-0344-8

**Published:** 2015-12-14

**Authors:** Mischa T. Rijnierse, Cornelis P. Allaart, Paul Knaapen

**Affiliations:** Department of Cardiology and Institute for Cardiovascular Research (IcaR-VU), VU University Medical Center, De Boelelaan 1117, 1081 HV Amsterdam, The Netherlands

**Keywords:** SPECT, PET imaging, computed tomography (CT), magnetic resonance imaging, ventricular tachycardia

## Abstract

Life-threatening ventricular arrhythmias (VA) are a major cause of death in patients with cardiomyopathy. To date, impaired left ventricular ejection fraction remains the primary criterion for implantable cardioverter-defibrillator therapy to prevent sudden cardiac death. In recent years, however, advanced imaging techniques such as nuclear imaging, cardiac magnetic resonance imaging, and computed tomography have allowed for a more detailed evaluation of the underlying substrate of VA. These imaging modalities have emerged as a promising approach to assess the risk of sudden cardiac death. In addition, non-invasive identification of the critical sites of arrhythmias may guide ablation therapy. Typical anatomical substrates that can be evaluated by multiple advanced imaging techniques include perfusion abnormalities, scar and its border zone, and sympathetic denervation. Understanding the principles and techniques of different imaging modalities is essential to gain more insight in their role in identifying the arrhythmic substrate. The current review describes the principles of currently available imaging techniques to identify the substrate of VA.

## Introduction

Sudden cardiac death (SCD) is a major health problem as it accounts for half of total cardiac mortality with an annual incidence of approximately 100 per 100.000 in the general population.^1^,^2^ In most cases, SCD is caused by ventricular arrhythmia (VA) in patients who suffer from underlying structural heart disease including ischemic cardiomyopathy, dilated cardiomyopathy, and hypertrophic cardiomyopathy.^1^ Implantable cardioverter-defibrillators (ICDs) have led to a significant reduction in mortality and have become standard clinical practice for the primary prevention of SCD in patients with left ventricular ejection fraction (LVEF) <30-35%, depending on cardiomyopathy etiology and heart failure symptoms.^3^-^5^ There are, however, several downsides of patient selection according to LVEF only. Recent studies have revealed that only 11-35% of patients with ICDs implanted for primary prevention of SCD actually receive appropriate ICD therapy in the ensuing years.^6^-^8^ It is clear that impairment of global LVEF is merely an indirect and aspecific risk factor for SCD, which is actually the result of underlying structural abnormalities causing electrical instability. A more detailed evaluation of the anatomical substrate of arrhythmias has therefore been an emerging topic of research over the last decade. Advanced cardiac imaging modalities such as nuclear imaging, cardiac magnetic resonance (CMR) imaging, and computed tomography (CT) hold great promise in evaluating the arrhythmic substrate to enhance risk stratification.^9^-^11^ More recently, these imaging modalities have also been used to identify critical sites of ventricular tachycardia (VT) in order to guide electrophysiological ablation therapy.^12^-^17^ It is therefore important to understand the principles of these imaging techniques used to identify the arrhythmic substrate. This review discusses the principles and techniques of currently available advanced imaging techniques in identifying the substrate for ventricular arrhythmias.

## Imaging Targets of the Arrhythmic Substrate

**Figure 1:**
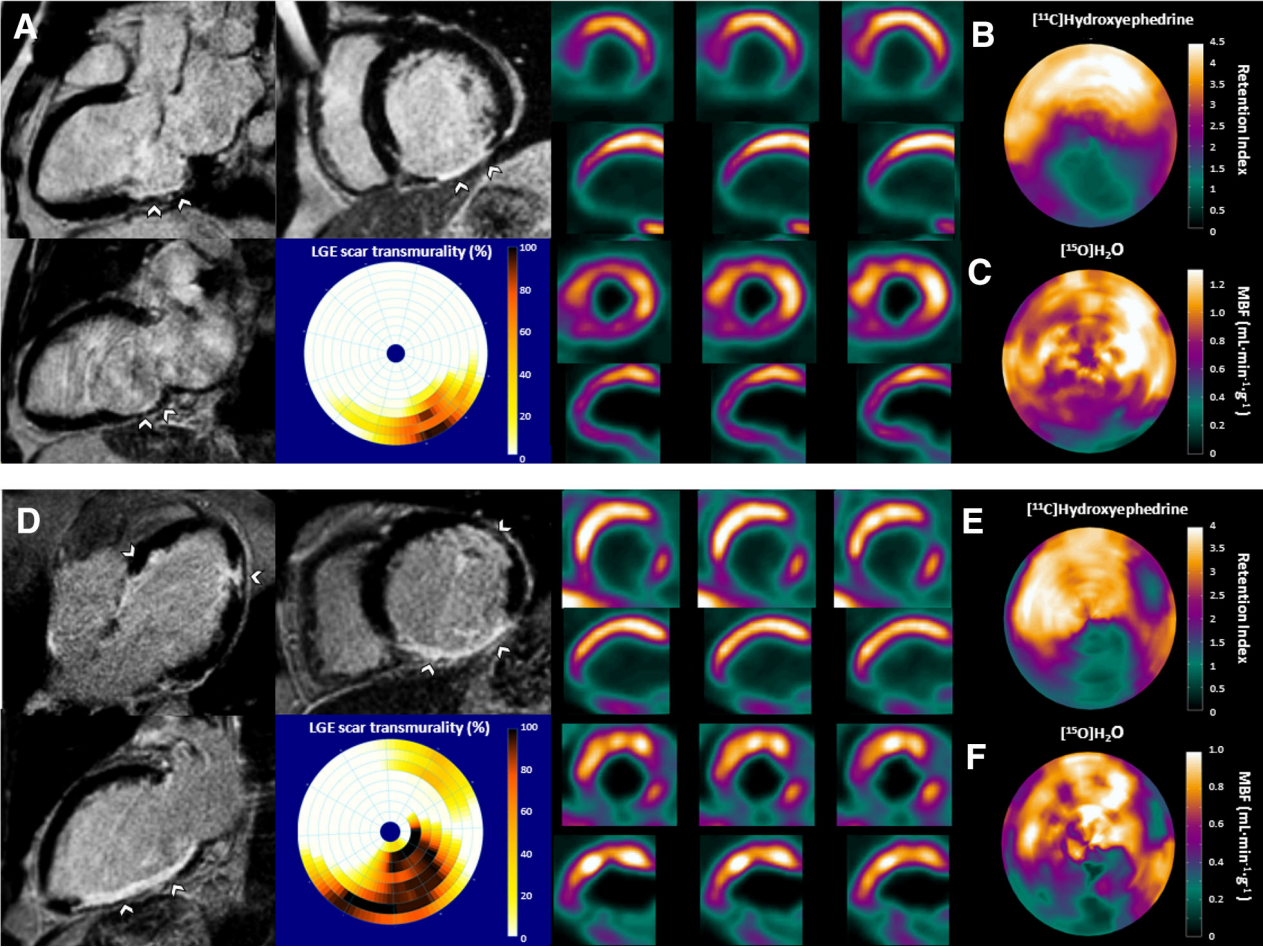
Imaging targets of the potential arrhythmic substrate in two patients with ischemic cardiomyopathy who were referred for ICD implantation for primary prevention of sudden cardiac death. [^15^O]H_2_O PET, [^11^C]HED PET, and LGE-CMR of patient 1 (**A**-**C**) displayed a basal inferior wall myocardial infarction with contrast enhancement in this region (**A**) and a corresponding perfusion defect (**C**) with a large innervation defect (**B**) that exceeded the infarct size, resulting in a significant innervation-perfusion mismatch. Patient 2 (**D**-**F**) showed a large inferior wall myocardial infarction with transmural contrast enhancement as well as subendocardial contrast enhancement at the anterolateral wall (**D**) at LGE-CMR. [^15^O]H_2_O PET and [^11^C]HED PET displayed corresponding perfusion and innervation defects with only limited innervation-perfusion mismatch. *CMR*, cardiovascular magnetic resonance; *LGE*, late gadolinium enhancement; *PET*, positron emission tomography

The pathophysiology of VA is complex and multifactorial. Traditionally, they are believed to be the result of a complex interplay between an anatomical substrate and transient triggers leading to electrical instability including increased automaticity, triggered activity, and re-entry.^18^ In more than 75% of SCD cases in the Western world, ischemic heart disease is responsible for the underlying arrhythmic substrate.^1^ Frequent culprits of susceptibility for VA are the presence of myocardial ischemia, scar burden, and sympathetic denervation.^19^ Various non-invasive imaging modalities have the potential to target these pathophysiological processes (Figure 1). Perfusion abnormalities may induce ischemia during an acute coronary syndrome or in the setting of chronic ischemic heart disease. In both scenarios, variations in myocardial perfusion may result in phases of ischemia and reperfusion which are associated with triggered activity and re-entry.^19^-^21^ The relation between ischemia and VA has been demonstrated in clinical studies.^22^,^23^ Nuclear imaging and CMR are considered to be the mainstay in perfusion imaging and therefore can be performed to identify the role of ischemia in the VA substrate. After myocardial infarction, inexcitable scar tissue is formed, surrounded by a border zone area with a mixture of viable myocytes and non-viable fibrous tissue. Consequently, this area comprises the fundamentals of a re-entry arrhythmia; heterogeneous prolonged conduction within the border zone adjacent to non-conduction scar tissue.^24^,^25^ This well-known theory has been supported by clinical studies demonstrating the association between the extent of scarring and susceptibility for VA.^26^ In fact, the infarct borderzone is generally considered as the pivotal part of the re-entry circuit.^27^ Furthermore, residual perfusion abnormalities in the infarct border zone may contribute to the arrhythmic vulnerability.^28^ Late gadolinium enhanced (LGE)-CMR is the preferred modality for scar and border zone visualization with high spatial resolution. Myocardial infarction does not merely result in a loss of myocytes but causes injury of cardiac sympathetic nerves to an even greater extent as nerve endings are more vulnerable to ischemia.^29^ Areas of denervated but viable myocardium are characterized by prolonged refractory periods and supersensitivity to catecholamines that cause increased heterogeneity in repolarization.^30^-^33^ Localized regions of hyperinnervation (nerve sprouting) due to increased heterogeneous post-injury regeneration have been observed in areas surrounding scar, which may further augment the electrical instability.^34^,^35^ Nuclear imaging is able to visualize cardiac sympathetic nerve function to assess the role of cardiac denervation. Finally, extensive scarring can initiate structural remodelling of the left ventricle (LV) resulting in loss of function, geometrical alterations, and increased wall stress. These structural changes itself have pro-arrhythmogenic effects due to modulation of action potential duration, calcium handling, and conduction.^36^,^37^

## Nuclear Imaging

**Figure 2:**
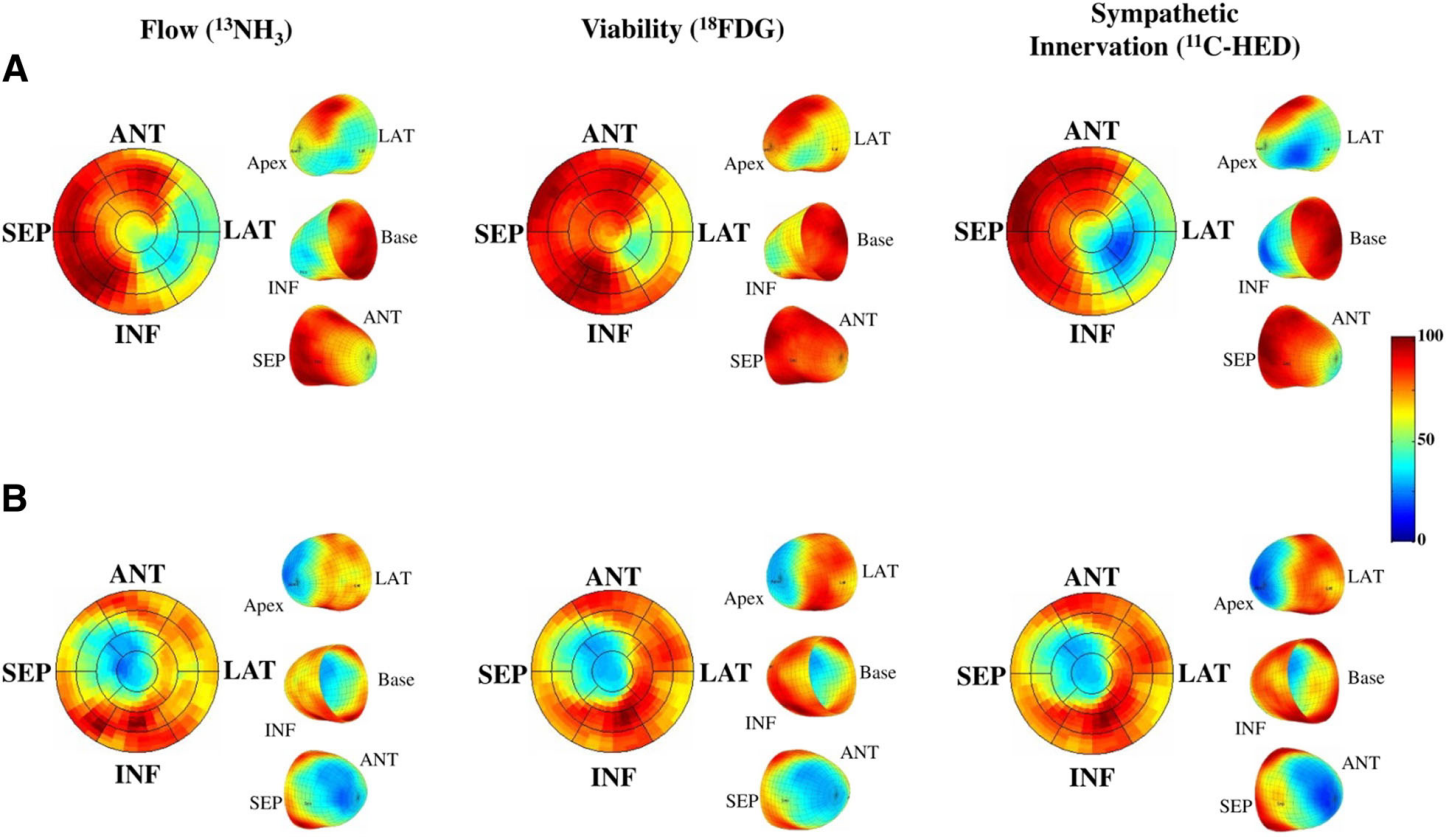
Examples of PET assessed perfusion, sympathetic innervation, and metabolism using different PET tracers in two patients with ischemic cardiomyopathy who were referred for primary prevention ICD therapy. (**A**) PET imaging revealed a mismatch between defects of perfusion and viability (infarct size), reflecting hibernating myocardium, as well as a mismatch between sympathetic innervation defect and infarct size, reflecting denervated but viable myocardium. This patient experienced appropriate ICD discharge for a fast ventricular tachyarrhythmia in follow-up. (**B**) PET imaging revealed matched defects of perfusion, viability, and sympathetic innervation. *ANT*, anterior; *ICD*, implantable cardioverter-defibrillator; *INF*, inferior; *LAT*, lateral; *PET*, positron emission tomography; *SEP*, septum. Reprint with permission^63^

Nuclear imaging provides the unique ability to target different pathophysiological aspects of the arrhythmic substrate, depending on the biological pathway of the utilized tracer. Consequently, nuclear techniques such as single photon emission computed tomography (SPECT) and positron emission tomography (PET) allow the evaluation of perfusion, scar, metabolism, and sympathetic innervation (Figure 2). Aspects of SPECT and PET techniques and image analysis with regard to the evaluation of the arrhythmic substrate are discussed below.

### Myocardial Perfusion Imaging

**Figure 3:**
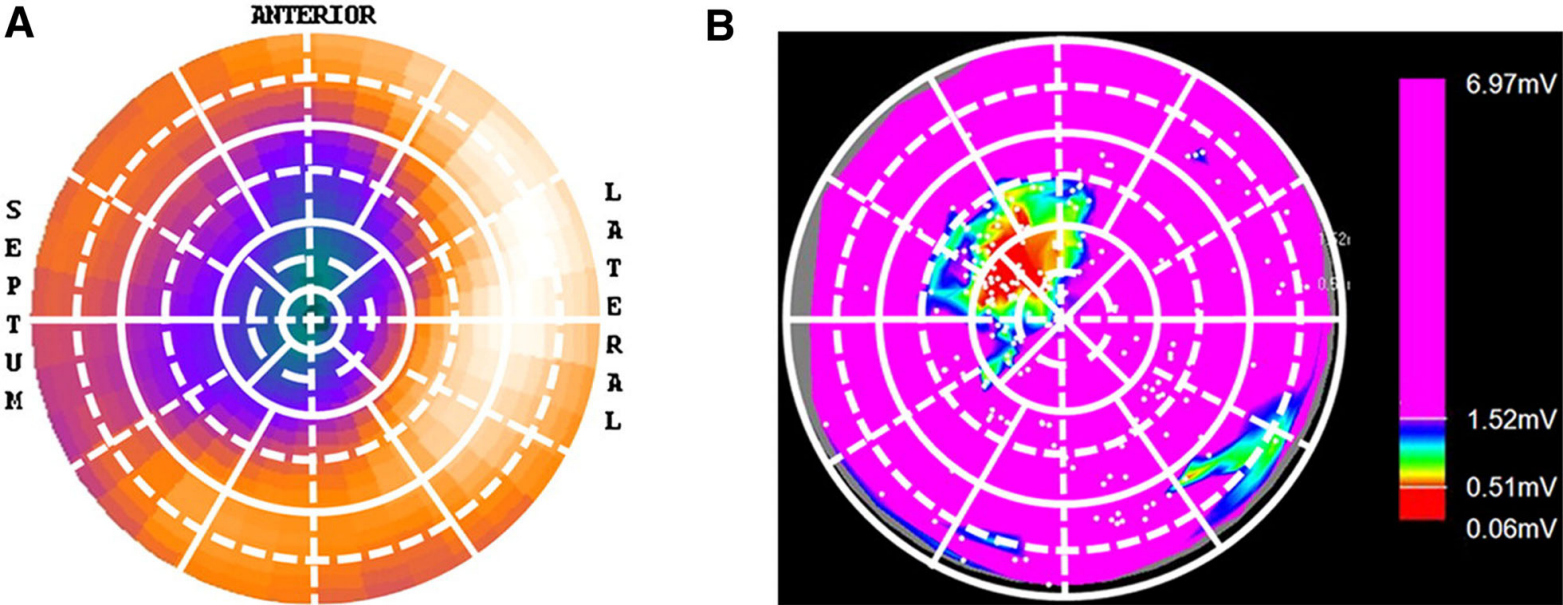
Example of ^201^TI SPECT assessed perfusion polar map (**A**) and corresponding electroanatomic voltage map (**B**) in a patient with ischemic cardiomyopathy. (**A**) SPECT polar map demonstrates anteroseptal and apical perfusion deficit which corresponded with the anteroseptal low-voltage scar area (red) as revealed by the corresponding voltage map (**B**). SPECT, single-photon emission computed tomography. Reprint with permission^15^

To date, the most widely used nuclear imaging technique to evaluate the extent and location of myocardial perfusion abnormalities is SPECT. Frequently used SPECT tracers in clinical practice include ^201^TI, [^99m^Tc]tetrofosmin, and [^99m^Tc]sestamibi. Based on relative tracer uptake in the myocardium during resting conditions and exercise or pharmacologically induced stress, information is obtained on the size of resting perfusion defects (reflecting scar or hibernating myocardium), stress perfusion defects (reflecting scar, hibernating myocardium and/or ischemia), and their reversibility (reflecting ischemia).^38^,^39^ Observational studies have demonstrated that the inducibility of VA during an electrophysiological study is related to the presence and size of resting perfusion defects in patients with ischemic cardiomyopathy.^40^,^41^ Moreover, inducible VTs were found to originate close to the border of resting perfusion defects as observed by McFarland et al,^42^ highlighting the importance of infarct border zone. These results were confirmed in a recent study by Tian et a^15^ who reconstructed 3D scar models obtained from ^201^TI SPECT to identify voltage-defined scar, border zone, and normal myocardium during electroanatomic mapping in order to guide VT ablations. It was demonstrated that impairment of ^201^TI uptake could accurately predict endocardial voltage-defined scar, which was the site of successful ablation in all patients (Figure 3). Most critical sites of VT were located within 1 cm of the SPECT assessed scar border, although the degree of ^201^TI uptake was not able to distinguish voltage defined scar and border zone. This may be the result of the relatively low spatial resolution of SPECT, limiting its value in border zone assessment. Nonetheless, a more recent retrospective analysis by Zhou et al,^43^ proposed a novel method of scar border zone evaluation using ^99m^Tc SPECT by calculating the size of myocardium with uptake between 40% and 60% of peak myocardial uptake surrounding the scar core area (defined as <40% of peak uptake) after comparing with template scans of healthy control patients. Interestingly, SPECT assessed border zone was shown to be significantly larger in patients who showed inducible sustained VA during an electrophysiological study as compared with non-inducible patients. The study, however, did not assess the site of origin of the induced VAs.^43^ Further studies are needed to evaluate the role of SPECT assessed border zone in identifying the arrhythmic substrate.

Residual ischemia after myocardial infarction may contribute to the electrical instability of the myocardium. This was revealed in a study by Paganelli et al^44^ who found a significant relation between the inducibility of VA and the presence and extent of residual ischemia as evaluated by stress-rest ^201^TI SPECT, in particular within the infarct area. No differences in fixed perfusion defects were observed, suggesting that residual ischemia might lead to additional vulnerability to VA.

**Figure 4:**
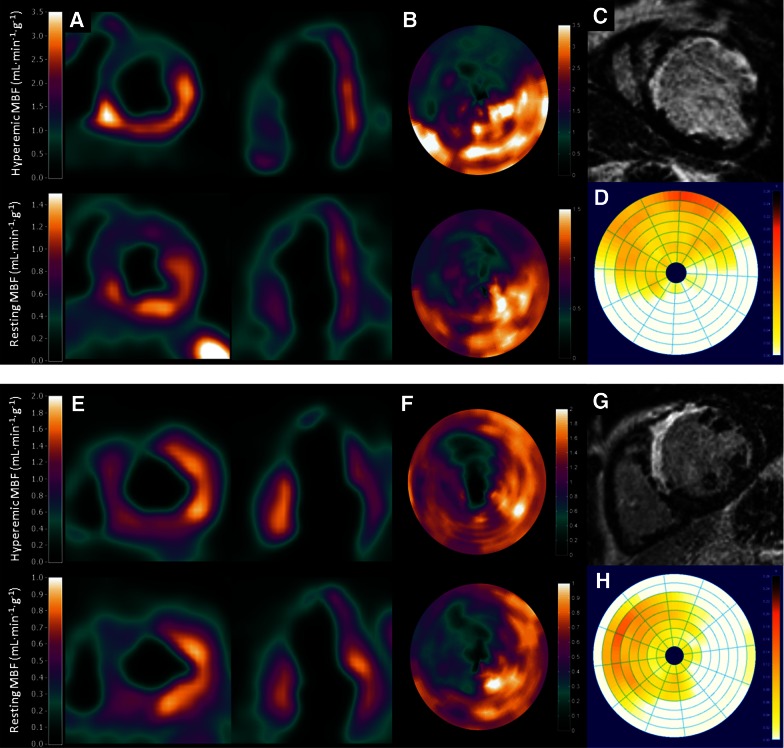
Examples of [^15^O]H_2_O PET and LGE-CMR scan in two patients with a history of an anterior wall myocardial infarction. Both patients displayed significant scar size (**C**, **D**, **G**, and **H**) and perfusion defect (**A**, **B**, **E** and **F**) in the anteroseptal wall. However, quantitative MBF results showed significantly impaired hyperemic MBF in patient 2 as compared with patient 1. Note the difference in scaling on the PET images (**A**, **B**, **E**, and **F**) between both patients. Electrophysiological study showed no inducible ventricular arrhythmias in patient 1 (**A**-**D**) whereas in patient 2 (**E**-**H**) a monomorphic ventricular tachycardia was induced. *CMR*, cardiovascular magnetic resonance; *LGE*, late gadolinium enhancement; *MBF*, myocardial blood flow; *PET*, positron emission tomography. Reprint with permission^28^

For detecting perfusion abnormalities, PET provides advantages over SPECT including higher spatial resolution and the ability of absolute quantification of myocardial blood flow (MBF). Furthermore, commonly used PET perfusion tracers such as [^15^O]H_2_O, [^13^N]NH_3_, and ^82^Rb have a higher extraction fraction and short physical half-life.^45^ As a result, more subtle regional perfusion abnormalities can be detected and quantified in mL·min^−1^·g^−1^. Of the available PET tracers, [^15^O]H_2_O is considered the most ideal tracer for flow quantification as it is freely diffusible and metabolically inert with an extraction of 100% from arterial blood, whereas ^82^Rb and [^13^N]NH_3_ are actively transported across the cell membrane with incomplete extraction and subsequently are metabolically trapped.^46^ Consequently, [^15^O]H_2_O PET quantifies MBF in viable tissue which is capable of exchanging water rapidly.^47^-^49^ This unique tracer characteristic might especially be of interest to assess subtle perfusion abnormalities in small areas of viable tissue within the infarct and border zone area which could provide more insight into the substrate and potential triggers of VA. A recently published pilot study demonstrated that impaired global hyperemic perfusion and coronary flow reserve as quantified by [^15^O]H_2_O PET were significantly related with VA inducibility during an electrophysiological study in 30 patients with ischemic cardiomyopathy who were referred for ICD implantation (Figure 4).^28^ These results need to be confirmed in larger studies to assess the relation between quantitative PET perfusion imaging and the VA substrate.

### Myocardial Metabolism

**Figure 5:**
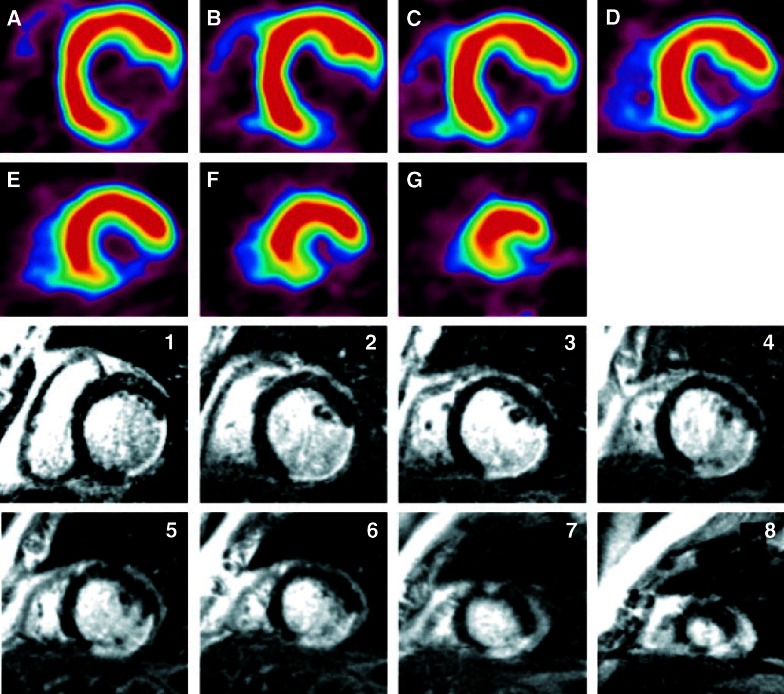
Example of [^18^F]FDG PET and LGE-CMR in a patient with a posterolateral myocardial infarction. The [^18^F]FDG PET images (**A**-**G**, base to apex) revealed a defect in the posterolateral wall extending to the inferior wall at the midventricular level. LGE-CMR (1-8, base to apex) shows transmural enhancement of a similar size in the same location. *CMR*, cardiovascular magnetic resonance; *LGE*, late gadolinium enhancement; *PET*, positron emission tomography. Reprint with permission^53^

Nuclear metabolic imaging to evaluate myocardial viability is commonly assessed using [^18^F]fluorodeoxyglucose (FDG) PET in clinical practice. [^18^F]FDG is a glucose analogue which is taken up into the cardiomyocytes by glucose transporters and subsequently become trapped after being metabolized. Preserved [^18^F]FDG uptake is considered a highly reliable marker for viable myocardium in patients with ischemic heart disease whereas severely reduced [^18^F]FDG uptake indicates non-viable scar tissue.^50^ The assessment of scar tissue using [^18^F]FDG PET is well validated^51^,^52^ and good correlations have been observed with LGE-CMR assessed fibrosis^53^,^54^ (Figure 5). As such, it provides a valid approach to visualize the size and location of non-viable scar in order to evaluate the site of the arrhythmic substrate although it has not been studied frequently for this purpose. Nonetheless, a study performed by Dickfeld et al^16^ demonstrated that 3D scar maps as assessed using hybrid [^18^F]FDG PET/CT fusion imaging could be integrated into clinical mapping system to visualize the 3D arrhythmogenic substrate in patients who were referred for VT ablation. A cut-off value of <50% [^18^F]FDG activity predicted voltage defined myocardial scar with a sensitivity and specificity of 89 and 93%, respectively, whereas voltage defined infarct borderzone contained a mean metabolic activity of 67±15%. These results imply that scar evaluation with [^18^F]FDG PET is able to identify critical arrhythmogenic substrates and may assist VT ablation therapies.^55^,^56^

When combined with perfusion imaging, viable myocardium can be further differentiated into the presence of subendocardial scar (mildly reduced [^18^F]FDG uptake and perfusion), hibernation (enhanced [^18^F]FDG uptake but impaired perfusion), and normal viable myocardium (preserved [^18^F]FDG and perfusion). The detection of increased uptake in hibernating myocardium is based on a metabolic shift from fatty acid to glucose consumption as the myocytes are hypoperfused and become dependent on anaerobic metabolism.^57^ Chronic repetitive ischemia in hibernating myocardium may lead to electrical remodeling due to adaptive responses of the myocytes which can contribute to VA susceptibility.^58^ Preclinical studies suggest that this process consists of cellular hypertrophy, altered calcium handling, and prolongation of action potentials leading to triggered activity and heterogeneous repolarization.^58^-^61^ Clinical studies, however, have reported inconsistent results linking the amount of hibernation to the actual occurrence of VA in follow-up.^62^,^63^

### Sympathetic Innervation

Targeting the cardiac sympathetic innervation using nuclear imaging has become an emerging approach to visualize the VA substrate. As discussed, the sympathetic nervous system may give rise to VA in several pathophysiological pathways including increased global sympathetic activity and regional cardiac sympathetic denervation resulting from ischemia, hibernation, or infarction.^64^ The mainstay of innervation imaging is based on the uptake, storage, and release of norepinephrine (NE), which is mediated by presynaptic sympathetic nerve terminals in response to variations of sympathetic drive. The sympathetic neurotransmitter NE is produced within sympathetic nerve terminals and stored in vesicles. After sympathetic activation, it is released into the sympathetic nerve cleft where it is able to bind on beta-adrenergic receptors of myocytes to transmit their sympathetic effects.^65^ Most of the NE, however, is cleared from the synaptic cleft, either by presynaptic neuronal re-uptake (uptake-1 mechanism), or by non-neuronal uptake by post synaptic transporters on myocytes (uptake-2 mechanism).^66^,^67^ After presynaptic re-uptake, NE is metabolized or stored back into the vesicles. Dependent on specific characteristics of the tracer used, several biological aspects of the neuronal function can be visualized. To date, most commonly used tracers are NE analogues [^123^I]metaiodobenzylguanide (MIBG) for SPECT and [^11^C]hydroxyephedrine (HED) for PET. Both tracers are resistant to metabolic enzymes and show high affinity for presynaptic NE uptake-1 allowing the visualization of presynaptic sympathetic nerve function.^65^,^68^ Other presynaptic PET tracers include [^11^C]epinephrine, [^11^C]phenylephrine, and ^18^F-LMI1195 while [^11^C]-CGP12177 is the most commonly used tracer for postsynaptic beta-adrenergic receptors.^65^,^69^

**Figure 6:**
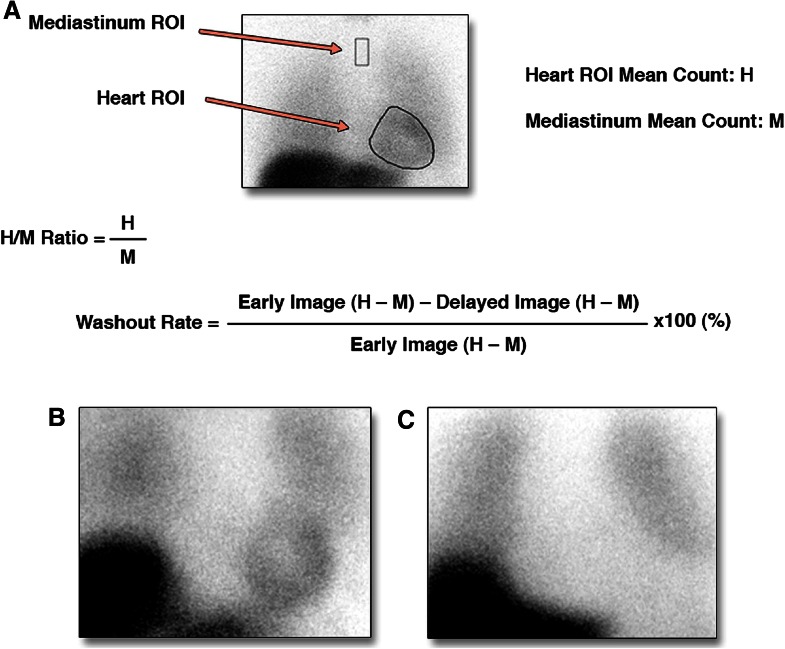
Example of global [^123^I]MIBG H/M ratio and wash-out rate calculation on planar [^123^I]MIBG imaging (**A**), and examples of normal cardiac [^123^I]MIBG activity in a patient with H/M ratio of 1.80 (**B**) compared with a [^123^I]MIBG scan showing a significantly impaired [^123^I]MIBG activity with H/M ratio of 1.10 (**C**). H/M, heart-to-mediastinum. Reprint with permission^70^

[^123^I]MIBG SPECT typically includes the assessment of early and delayed planar as well as tomographic images at 15-30 minutes and 3-4 hours after tracer injection, respectively. In planar images, global myocardial uptake is evaluated by calculating the heart to mediastinum (H/M) uptake ratio. Furthermore, [^123^I]MIBG washout rate (WR) is defined as the difference between the early and delayed H/M ratios and primarily reflects presynaptic NE turnover, a marker for sympathetic activity (Figure 6).^70^ In patients with cardiomyopathy, increased sympathetic drive causing high nerve firings and insufficient re-uptake is reflected by a decrease in delayed H/M ratio and increased WR.^68^ Assessment of size and location of regional innervation defects on tomographic delayed [^123^I]MIBG images allow more detailed evaluation of the anatomic substrate. Accordingly, Klein et al^17^ recently acquired 3D reconstructions of [^123^I]MIBG SPECT and combined these innervation maps with endocardial voltage maps as assessed during electrophysiological study in patients with ischemic cardiomyopathy who were referred for VT ablation. Areas of cardiac denervation were found to be significantly larger as compared with voltage defined scar reflecting the higher vulnerability of cardiac nerves to ischemia. More importantly, all successful ablation sites were located in myocardium with abnormal [^123^I]MIBG uptake whereas in 36% these sites were found in normal myocardium when assessed with voltage mapping. These results indicate the role of sympathetic denervation as VA substrate, which may be more important than scar. When combined with SPECT perfusion imaging, regional areas of viable but denervated myocardium can be detected which are potential sites of electrical instability.^71^ In a pilot study, Bax and colleagues performed [^123^I]MIBG and [^99m^Tc]tetrofosmin SPECT in patients with ischemic cardiomyopathy who underwent electrophysiological testing. Patients with inducible VA showed larger areas of [^123^I]MIBG defects obtained from delayed SPECT images. The mismatch size, however, between innervation and perfusion defects was not related to VA susceptibility.^72^ As areas of denervated but viable myocardium may be intertwined with the infarct border zone which often contains critical sites of VA, the assessment of [^123^I]MIBG uptake in this area specifically may be of interest. Accordingly, in a post-hoc analysis of the previous mentioned study by Bax et al,[^123^I]MIBG uptake in the infarct border zone defined using [^99m^Tc]tetrofosmin SPECT predicted the inducibility of sustained VA during electrophysiological testing with higher accuracy compared with scar or scar border zone itself.^43^

**Figure 7:**
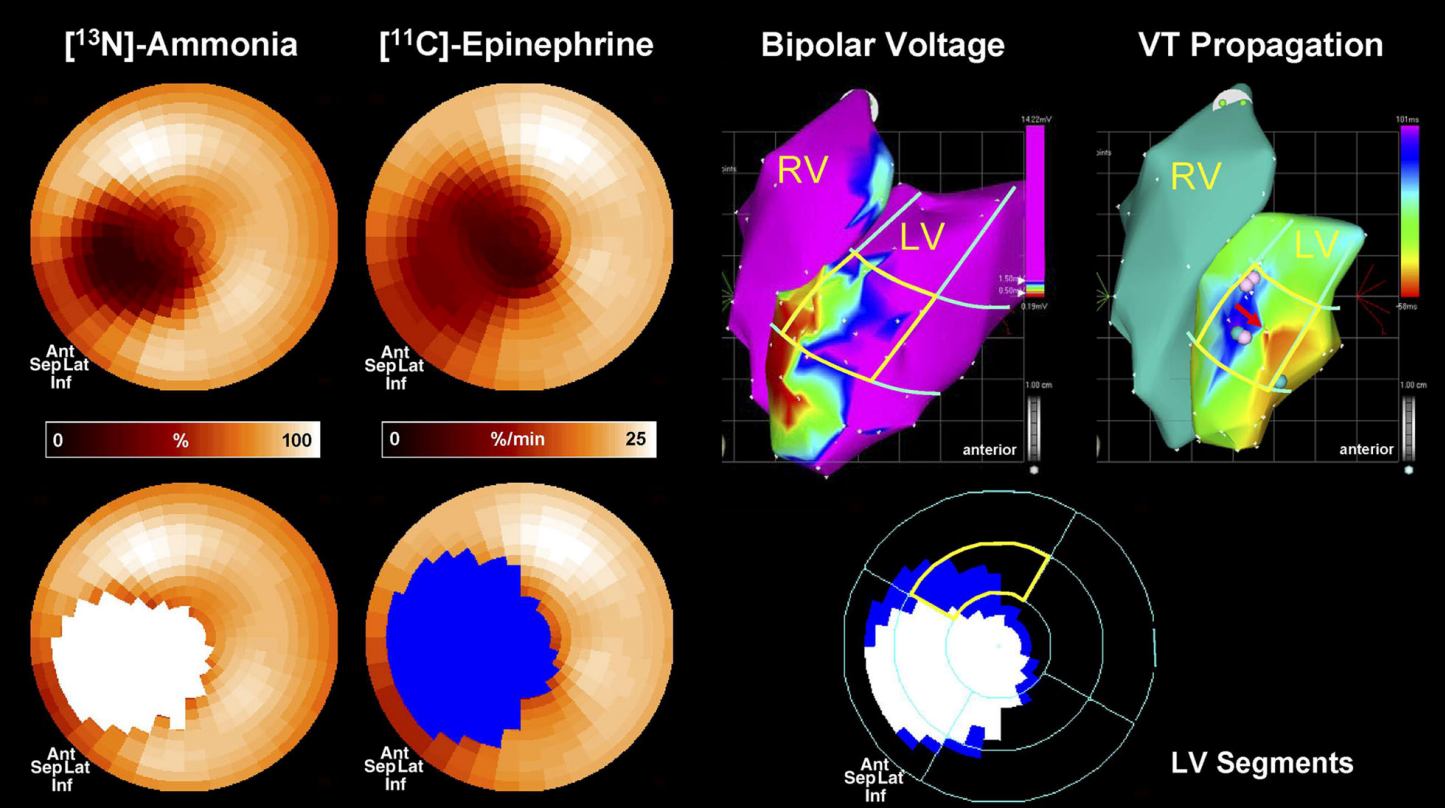
Electrophysiological study in an animal with inducible VT. PET assessed perfusion and innervation polarmaps demonstrated an innervation defect (*blue*) which exceeded the size of the perfusion defect (*white*), predominantly in the distal anterior wall segment (*bottom right*). Three-dimensional electroanatomic voltage maps (*top right*) demonstrated reduced voltage in apex and distal anterior septal wall and propagation map showed earliest activation of the VT in the infarct border zone in the distal anterior wall (*red arrow*), which corresponded with the location of the innervation-perfusion mismatch. *PET*, positron emission tomography; *VT*, ventricular tachycardia. Reprint with permission^78^

**Figure 8:**
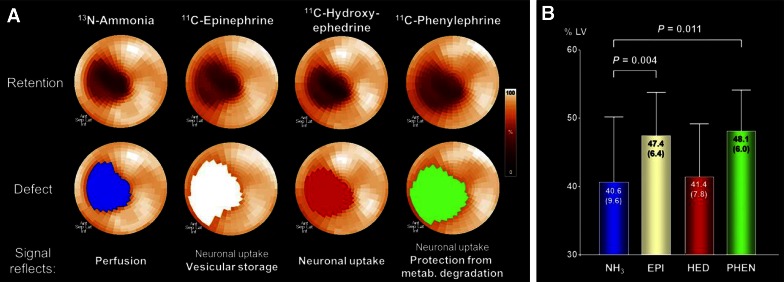
PET assessment of myocardial innervation using multiple radiolabeled catecholamine analogues in an animal with myocardial infarction. (**A**) Polar maps of representative infarct animal showing retention of 4 tracers (*top*), along with color-coded defect areas. Epinephrine and phenylephrine defects exceeded the perfusion defect, whereas hydroxyephedrine defect is comparable to the perfusion defect. (**B**) Bar chart showing group results of defect sizes. *Ant*, anterior; *EPI*, epinephrine; *HED*, hydroxyephedrine; *Inf*, inferior; *Lat*, lateral; *metab.*, metabolic; *NH3*, ammonia; *PET*, positron emission tomography; *PHEN*, phenylephrine; *sep*, septal. Reprint with permission^79^

**Figure 9:**
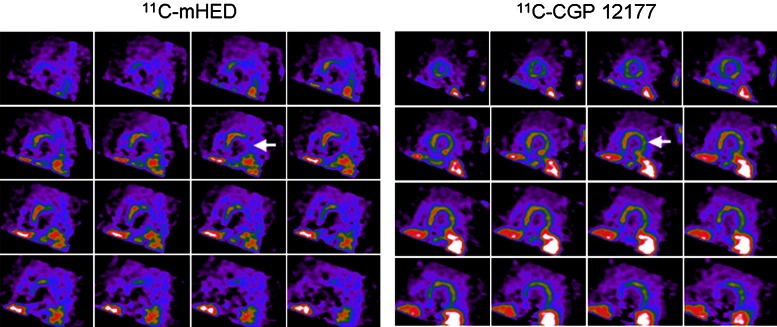
PET imaging of presynaptic sympathetic nerves using [^11^C]HED and postsynaptic beta-adrenergic receptors using [^11^C]CGP12177 in a patient with ischemic cardiomyopathy. White arrows indicate extensive mismatch between presynaptic innervation and postsynaptic beta-adrenergic receptors in the lateral wall. *HED*, hydroxyephedrine; *PET*, positron emission tomography. Reprint with permission^80^

The assessment of sympathetic innervation using PET may have incremental value in evaluating the arrhythmic substrate. First, the superior spatial resolution of PET results in more detailed assessment of regional sympathetic innervation and innervation/perfusion mismatch areas. Second, dynamic imaging protocols enables absolute quantification of sympathetic nerve retention of tracers.^73^ Third, the availability of multiple tracers, each acting on different biological aspects of the nerve terminals or beta-adrenergic receptors, provides further insight into the cardiac neuronal function.^74^ The [^11^C]HED PET protocol typically consists of a dynamic scan with a duration of 40-60 minutes after injection of the tracer. Subsequently, sympathetic nerve uptake is frequently quantified using the retention index, which is defined as the uptake in the final frame of the scan divided by the integral of the arterial input curve.^75^ In addition, kinetic modelling of the tracer may be performed which results in more absolute quantification of the volume of distribution of the tracer and its wash-out.^73^ Although [^11^C]HED retention, washout and defect size are closely related to [^123^I]MIBG SPECT variables, the ability of quantification may overcome problems with relative defect size calculations such as global downregulation in patients with heart failure.^76^ In addition, by calculating the influx rate (K_1_) of [^11^C]HED, Harms et al recently demonstrated the feasibility of assessing perfusion and innervation defects to evaluate mismatch areas using a single dynamic [^11^C]HED PET scan.^77^ Several studies have showed altered electrophysiological characteristics within areas of reduced [^11^C]HED uptake including prolonged refractoriness and supersensitivity to catecholamines.^30^,^33^ Sasano et al^78^ showed that the extent of viable but denervated myocardium quantified with [^11^C]epinephrine and [^13^N]NH_3_ PET was associated with inducible VTs in a porcine model. Importantly, the area of denervated but viable myocardium was related to the site of initiation of the induced VTs as well as decreased endocardial voltage obtained by voltage mapping (Figure 7). Recently, the uptake of multiple presynaptic tracers was explored in viable but devervated myocardium in a similar porcine myocardial infarction model.^79^ While [^11^C]epinephrine retention was impaired in the infarct border zone, [^11^C]HED retention was relatively preserved, suggesting compromised vesicular storage rather than impaired uptake or complete denervation (Figure 8). In fact, immunohistology of explanted hearts showed intact nerve fibers and nerve sprouting in the infarct border zone.^79^ However, results from the PAREPET study confirmed the presence of larger [^11^C]HED assessed denervation areas as compared to perfusion defects in patients with ischemic cardiomyopathy, which may reflect a more advance state of neuronal impairment in chronic ischemic heart disease.^63^ Next to injured presynaptic sympathetic nerves, patients with ischemic cardiomyopathy show abnormalities of the postsynaptic beta-adrenergic receptors, although to a lesser extent (Figure 9). Consequently, a mismatch between presynaptic and postsynaptic adrenergic signaling is present which might be vulnerable to arrhythmias due to locally increased levels of catecholamines affecting repolarization.^80^. Finally, abnormal [^11^C]HED retention has been observed in non-ischemic cardiomyopathies^81^-^83^ as well as primary arrhythmogenic disorders.^84^-^86^

## Cardiac Magnetic Resonance Imaging

CMR imaging is increasingly used in daily clinical practice to quantify left ventricular volumes and function. Due to superior accuracy and reproducibility, CMR is considered as gold standard for the evaluation of LV dimensions.^87^ Furthermore, 3D evaluation of LV geometry may provide insight in remodelling and regional wall stress, which is linked to VA.^88^ In addition, contrast agents such as gadolinium enables qualitative and semi-quantitative assessment of perfusion based on the wash-in of the contrast agent into the myocardium. By comparing first-pass perfusion during pharmacologically induced stress and resting conditions, ischemia can be detected. However, no studies have evaluated the role of CMR perfusion imaging in identifying the substrate of VA to date. The actual superior value of CMR in identifying the arrhythmic substrate is the ability of scar tissue characterization using LGE which will be discussed below.

### Late Gadolinium Enhancement

**Figure 10:**
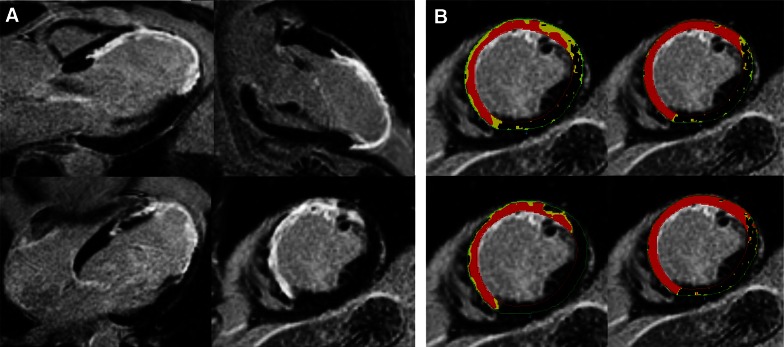
(**A**) Example of short and long axis LGE-CMR images in a patient with a large anterior wall myocardial infarction with contrast enhancement visible in the anteroseptal, anterior, and anterolateral wall. (**B**) Examples of several previously validated quantification methods of the scar core area (*red*) and scar border zone area (*yellow*), each resulting in significant a different scar core and border zone size

**Figure 11:**
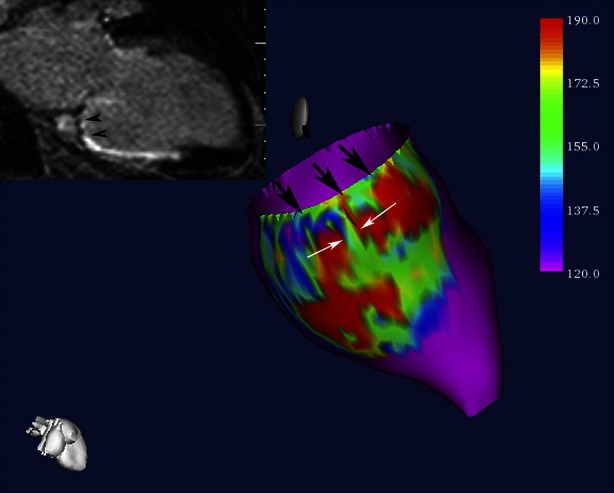
Example of signal intensity map of the subendocardial tissue projected onto a 3-dimensional endocardial shell reconstruction of the left ventricle. Scar core area with high signal intensity is presented in *red*, whereas normal myocardium is presented in *purple*. *Black* and *white arrows* indicate two corridors of continuous heterogeneous tissue with intermediate signal intensity (*green*) surrounded by scar tissue. The left upper corner shows the corresponding long-axis LGE-CMR image with heterogeneous tissue located between the mitral valve and the inferior scar (*black arrowheads*). *CMR*, cardiovascular magnetic resonance imaging; *LGE*, late gadolinium enhancement. Reprint with permission^102^

The visualization of fibrotic tissue is based on alterations of T1 relaxation characteristics after intravenous contrast administration. Gadolinium diffuses out of the intravascular volume and after 10-15 minutes it is distributed within the extracellular tissue as it cannot enter the myocytes. Hence, magnetic characteristics in fibrotic tissue are altered resulting in a relatively higher signal intensity (SI), visible as bright areas when compared to the normal remote myocardium on the T1-weighted images.^89^ Scar tissue assessment using LGE-CMR has a high spatial resolution and is well validated in histopathologic studies.^90^,^91^ LGE can be quantified in absolute grams of scar tissue using the full-with-at-half-maximum which defines scar core tissue as SI >50% of maximum SI in hyperenhanced area, or using different SD thresholds (>3-5 SD) of the mean SI in remote myocardium.^92^ In addition, areas of intermediate enhancement (‘the gray zone’) can be assessed separately using various SI thresholds such as 35-50% of maximum SI or 2-3 SD of mean remote SI to evaluate the scar border zone which contains a heterogeneity of viable and non-viable tissue^39^,^93^ These different methodologies of quantification will result in significantly different absolute grams of scar core and scar border zone as demonstrated in Figure 10, although all seem related to the VA substrate.^94^,^95^ Bello et al^26^ was the first to describe the association between LGE-CMR assessed total scar size and inducibility of sustained VA in patients with ischemic cardiomyopathy. Subsequently, Schmidt et al^96^ reported that quantification of the heterogeneous scar size resulted in improved prediction of VT inducibility. In addition, the presence of nonischemic fibrosis is associated with VT inducibility in patients with dilated cardiomyopathy.^97^ Although these studies suggest a direct link between LGE scar characteristics and the arrhythmogenic substrate, they did not compare the location of scar or borderzone and the site of origin of the induced VA. This was explored in detail by more recent studies who evaluated patients that underwent VT ablation. Comparable to the described PET and SPECT studies, 3D reconstructions of LGE-CMR assessed scar can be integrated into electroanatomic mapping systems to visualize the arrhythmic substrate which may assist VT ablation strategies.^12^,^13^,^98^-^103^ For this purpose, LGE-CMR might be superior compared with other investigated imaging modalities as it is able to discriminate between endocardial, midwall, and epicardial scar localizations, whereas invasive electroanatomic mapping only evaluates the endocardial or epicardial surface.^104^ By projecting the SI, transmural extension, or localization of scar acquired from consecutive LGE cine images onto 3D shells of the LV with coloured scales, 3D LGE maps are generated. Integration of 3D LGE with electroanatomic maps provide unique insight into LGE characteristics of critical sites of the VT. Desjardins et al^101^ showed that critical sites of postinfarction VT are located within areas of high contrast enhancement as assessed with LGE-CMR. In addition, Perez-David et al^102^ demonstrated that patients with ischemic heart disease and documented sustained VT frequently show continuous tissue corridors of border zone areas inside scar tissue on 3D endocardial SI maps (Figure 11). More importantly, these heterogeneous tissue corridors corresponded with the size and location of slow conduction channels as observed during voltage mapping and often were the critical isthmus site of VT. In another study using high resolution 3T LGE-CMR, 74% of the critical isthmus sites of VTs were identified by border zone channels which were more commonly seen in the endocardium.^98^ Other studies that integrated LGE-CMR in electroanatomic mapping demonstrated that critical sites of the VT circuit are often located very close to scar tissue with high transmural extension and/or near the transition of scar core and border zone.^12^,^99^,^100^,^104^ The described studies provide evidence for the relation of LGE-CMR assessed scar characteristics and the VA substrate, in particular the location of infarct border zone and more transmurally extended scar tissue. Finally, a preclinical study performed by Ng et al^105^ showed the potential of performing a non-invasive virtual electrophysiological study in an in-vivo 3D LGE-CMR model using a computer simulation after applying a mathematical model that predicts VT circuits. It was demonstrated that most of the virtually induced VTs using CMR corresponded with actual inducible VTs during invasive electrophysiological study.

### T1 Mapping

Several limitations of LGE-CMR should be acknowledged. First, standardization of imaging protocols and analyses is lacking as different SI thresholds are used for scar and border zone definitions. Second, LGE is based on relative differences in SI between fibrotic tissue and supposedly normal myocardium. Consequently, LGE is only able to detect focal areas of fibrotic tissue, whereas diffuse interstitial fibrosis cannot be identified. The occurrence of diffuse interstitial fibrosis, however, is not an infrequent pathological phenomenon in cardiomyopathy, not merely in nonischemic etiologies, but also in ‘remote’ areas of ischemic cardiomyopathy.^106^-^109^ More importantly, interstitial fibrosis may affect electrical propagation between myocytes due to alterations in cell-coupling, thereby contributing to the arrhythmic substrate.^110^ T1 mapping has emerged as a new CMR technique to evaluate diffuse fibrosis as it provides a pixel-wise quantification of myocardial T1 values, thereby overcoming the limitations of LGE-CMR. By quantifying absolute native pre-contrast T1 as well as post-contrast T1 values, the extracellular volume index can be calculated which is closely correlated with histopathologic measures of diffuse fibrosis.^106^,^108^ Although it has recently been demonstrated that absolute T1 values may be linked to the occurrence of VA in ischemic and non-ischemic cardiomyopathy, future studies are needed to confirm the role of diffuse fibrosis as assessed with T1 mapping in identifying the VA substrate^111^

## Computed Tomography

Contrast enhanced CT is an alternative modality for scar evaluation with high spatial and temporal resolution. Comparable to LGE-CMR, late enhancement CT can be acquired approximately 10 minutes after contrast injection with a standard single-energy or dual-energy cardiac CT. Both have been demonstrated to correlate well with LGE-CMR assessed scar as well as histopathologic findings^112^-^115^ In addition, perfusion defects obtained from qualitative first-pass CT perfusion imaging may provide information on the extent and location of scar. Few studies have assessed the role of contrast enhanced CT in identifying the arrhythmic substrate. Tian et al^14^ evaluated abnormalities in LV wall thickness, wall thickening, and perfusion using 3D contrast enhanced CT in 11 patients with ischemic cardiomyopathy who underwent VT ablation. Although all were related with abnormal endocardial voltage obtained from electroanatomic mapping, areas of CT hypoperfusion showed superior correlations with scar and border zone and represented the site of successful ablation in 82% of cases. It has been demonstrated that the size of both CT early hypoperfused and late hyperenhanced areas are comparable to LGE-CMR assessed scar size.^113^,^116^ Consequently, contrast enhanced CT may be used to identify the arrhythmic substrate in patients with ischemic cardiomyopathy. However, nephrotoxic effects of iodinated contrast and the occurrence of imaging artefacts resulting from metal devices may hinder its use in some cardiomyopathy patients.

## Conclusion

Advanced non-invasive imaging techniques such as nuclear imaging, CMR, and CT have emerged during the past decades to evaluate multiple aspects of the substrate of VA. Typical imaging targets include myocardial perfusion abnormalities, hibernation, scar, scar border zone, and sympathetic innervation. Their role in identifying the origin of VA has been shown in multiple studies suggesting significant value in diagnosis and risk assessment of VA, and a potential role in guiding ablation strategies. For detecting perfusion abnormalities, most experience has been gained using SPECT and PET, whereas LGE-CMR is the preferred modality for scar and border zone evaluation. More biological aspects of the arrhythmic substrate such as metabolism and sympathetic nerve function are well performed using PET. As targets of multiple imaging modalities may be intertwined, studies are warranted that compare imaging parameters of the arrhythmic substrate obtained by different modalities to define the preferred method to identify the substrate of VA.

## Abbreviations

CT: Computed tomography
FDG: Fluorodeoxyglucose
HED: Hydroxyephedrine
H/M: Heart to mediastinum
LGE-CMR: Late gadolinium enhanced-cardiac magnetic resonance imaging
LV: Left ventricle
LVEF: Left ventricular ejection fraction
MBF: Myocardial blood flow
MIBG: Metaiodobenzylguanide
NE: Norepineprhine
PET: Positron emission tomography
SCD: Sudden cardiac death
SI: Signal intensity
SPECT: Single-photon emission computed tomography
VA: Ventricular arrhythmia
VT: Ventricular tachycardia
WR: Washout rate
